# Supplementary material for: Epigenome-wide association study of human frontal cortex identifies differential methylation in Lewy body pathology
Source: Nat Commun. 2022 Aug 22;13:4932. doi: 10.1038/s41467-022-32619-z (PMC9395387; doi:10.1038/s41467-022-32619-z)
Supplement: Supplementary file 1 — Supplementary Information [file 41467_2022_32619_MOESM1_ESM.pdf]

Epigenome-wide association study of human frontal cortex identifies differential methylation in Lewy body pathology - Supplementary Information

Supplementary Table 1. **Sample demographics**

Dataset	Neuropathological diagnosis	N	Mean age at death	Min age	Max age	Prop. Female	Mean PMD min	Min PMD min	Max PMD min
Discovery sample NBB	Control	73	80.3	49	99	0.70	451	215	1335
	iLBD	29	85.9	72	98	0.52	400	150	705
	PD	139	77.7	56	96	0.35	360	170	770
	DLB	81	78.6	54	98	0.43	303	190	475
Replication sample BDR	Control	135	83.1	41	104	0.50	3193	390	9300
	iLBD	17	84.9	70	102	0.24	2715	540	7500
	LBD	48	83.3	61	97	0.44	3431	480	9180

PMD = Post mortem delay, NBB = Netherlands Brain Bank, BDR = UK Brains for Dementia Research, DLB = Dementia with Lewy Bodies, iLBD = incidental Lewy Body Disease, PD = Parkinson's disease, LBD = Lewy Body Disease (umbrella term covering Parkinson's disease and dementia with Lewy bodies)

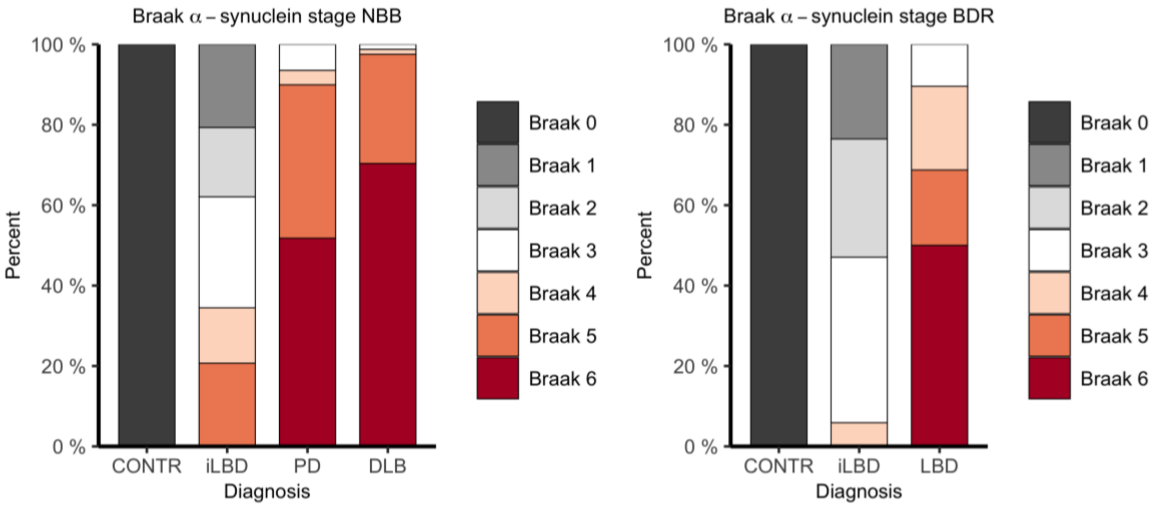

Supplementary Figure 1. **Distribution of Braak Lewy body stages across neuropathological diagnoses.** Distribution of Braak α-synuclein stages is shown for the NBB discovery (left) and BDR replication sample (right). See above for abbreviations.

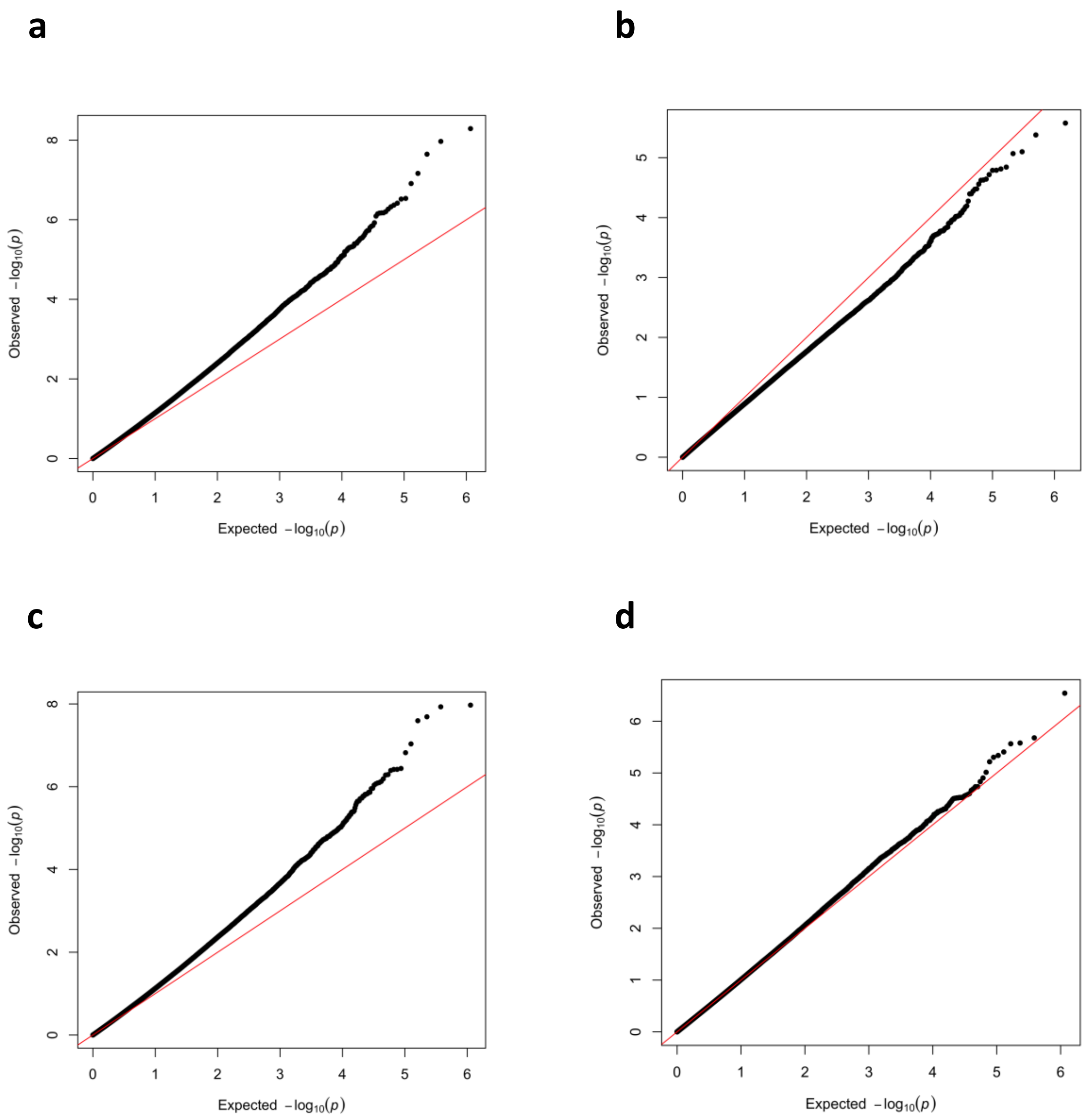

Supplementary Figure 2. **QQ plots.** Plots are based on two-sided p-values from **a** Primary linear regression analysis of the Netherlands Brain Bank (NBB) dataset (λ = 1.177), **b** Linear regression analysis of the Brains for Dementia Research (BDR) replication dataset ((λ = 0.85), **c** Fixed-effects meta-analysis of linear regression results from the NBB and BDR datasets (λ = 1.136) and **c** Mixed linear model-based omics analysis (MOA) as implented in the OSCA software package applied to NBB data (λ = 0.991).

a

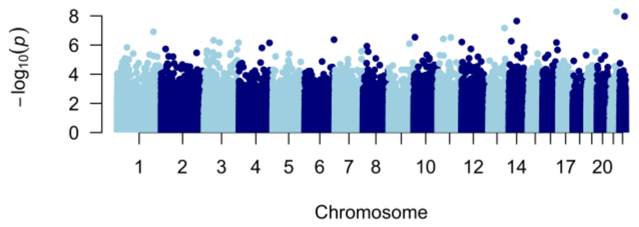

b

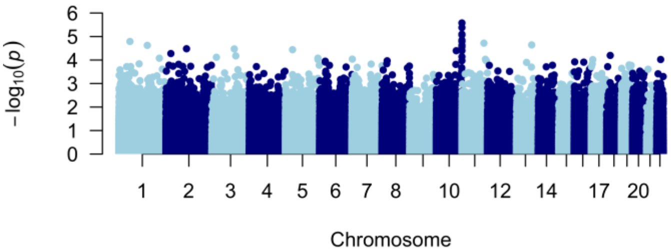

c

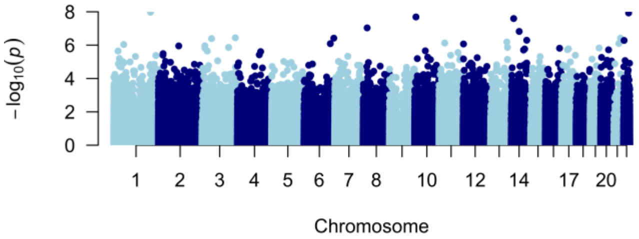

d

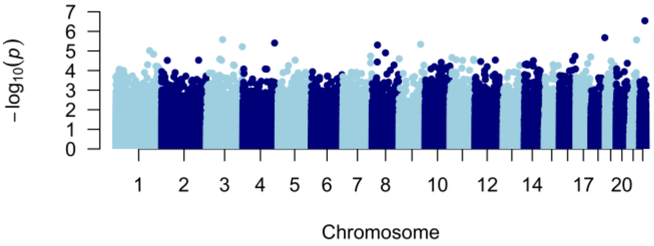

Supplementary Figure 3. **Manhattan plots.** Plots are based on two-sided p-values from **a** Primary linear regression analysis of the Netherlands Brain Bank (NBB) dataset, **b** Linear regression analysis of the Brains for Dementia Research (BDR) replication dataset (note the peak corresponding to the *CYP2E1* region), **c** Fixed-effects meta-analysis of NBB data and BDR linear regression results, and **d** Mixed linear model-based omics analysis (MOA) applied to NBB data.

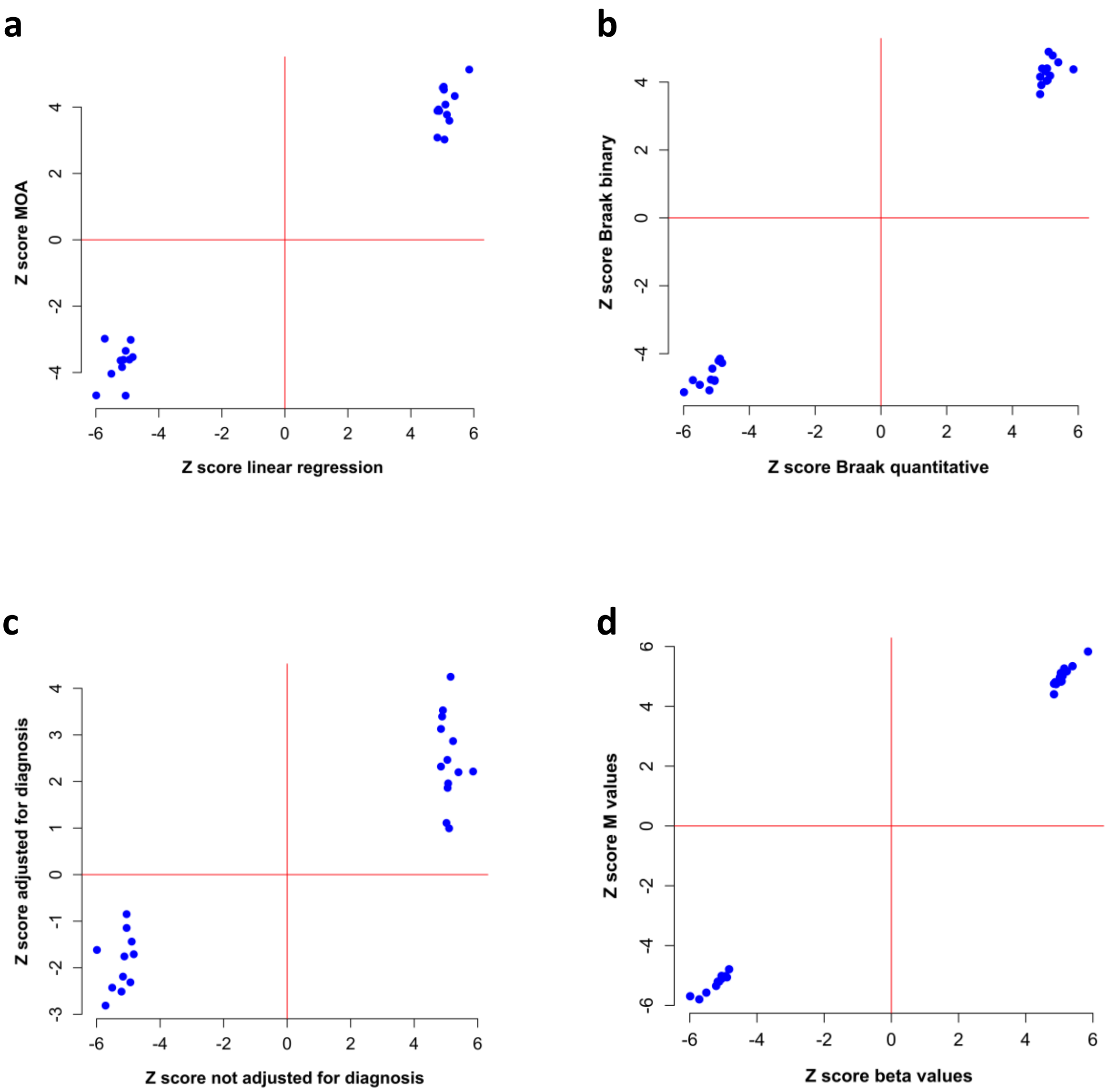

Supplementary Figure 4. **Comparing top probe Z scores across alternative analysis methods.** The plot shows data for the top 24 probes associated with Braak Lewy Body stage at FDR-corrected significance level in linear regression analysis of the Netherlands Brain Bank discovery data set. Z-scores were calculated as effect estimate divided by standard error. Plots compare the main linear regression model Z scores to **a** results from mixed linear model-based omics analysis (MOA) as implemented in the OSCA software package, **b** linear regression with Braak stage defined as a binary variable (stage 0-2 vs stage 3-6), **c** linear regression adjusting for neuropathological diagnosis as a covariate in the model and **d** linear regression using M values rather than methylation beta values as input in an otherwise identical model.
