# Supplementary material for: Epigenome-wide association study of human frontal cortex identifies differential methylation in Lewy body pathology
Source: Nat Commun. 2022 Aug 22;13:4932. doi: 10.1038/s41467-022-32619-z (PMC9395387; doi:10.1038/s41467-022-32619-z)
Supplement: Supplementary file 3 — Description of Additional Supplementary Files [file 41467_2022_32619_MOESM3_ESM.pdf]

Description of Additional Supplementary Files

File Name: Supplementary Data 1
Description: Results from linear regression of probes passing FDR significance in NBB discovery data, with BDR replication and fixed-effect meta-analysis

File Name: Supplementary Data 2
Description: Probes passing FDR threshold in meta-analysis with consistent direction of effect and $p < 0.05$ in both datasets

File Name: Supplementary Data 3
Description: Results from mixed linear model-based omics association (MOA) as compared to linear regression for 24 FDR-nominated probes

File Name: Supplementary Data 4
Description: Results from linear regression defining Braak Lewy body stage as binary (0-2 vs 3-6) compared to main model for 24 FDR-nominated probes

File Name: Supplementary Data 5
Description: Results from linear regression adjusting for neuropathological diagnosis compared to main model for 24 FDR-nominated probes

File Name: Supplementary Data 6
Description: Results from linear regression using M values compared to main anaylsis based on methylation beta values for 24 FDR-nominated probes
