# Supplementary material for: Epigenome-wide association study of human frontal cortex identifies differential methylation in Lewy body pathology
Source: Nat Commun. 2022 Aug 22;13:4932. doi: 10.1038/s41467-022-32619-z (PMC9395387; doi:10.1038/s41467-022-32619-z)
Supplement: Supplementary file 5 — Reporting Summary [file 41467_2022_32619_MOESM5_ESM.pdf]

nature portfolio

Corresponding author(s): Lasse Pihlstrøm

Last updated by author(s): Jul 27, 2022

Reporting Summary

Nature Portfolio wishes to improve the reproducibility of the work that we publish. This form provides structure for consistency and transparency in reporting. For further information on Nature Portfolio policies, see our Editorial Policies and the Editorial Policy Checklist.

Statistics

For all statistical analyses, confirm that the following items are present in the figure legend, table legend, main text, or Methods section.

n/a	Confirmed	
☐	☒	The exact sample size (*n*) for each experimental group/condition, given as a discrete number and unit of measurement
☐	☒	A statement on whether measurements were taken from distinct samples or whether the same sample was measured repeatedly
☐	☒	The statistical test(s) used AND whether they are one- or two-sided<br>*Only common tests should be described solely by name; describe more complex techniques in the Methods section.*
☐	☒	A description of all covariates tested
☐	☒	A description of any assumptions or corrections, such as tests of normality and adjustment for multiple comparisons
☐	☒	A full description of the statistical parameters including central tendency (e.g. means) or other basic estimates (e.g. regression coefficient) AND variation (e.g. standard deviation) or associated estimates of uncertainty (e.g. confidence intervals)
☐	☒	For null hypothesis testing, the test statistic (e.g. *F*, *t*, *r*) with confidence intervals, effect sizes, degrees of freedom and *P* value noted<br>*Give P values as exact values whenever suitable.*
☒	☐	For Bayesian analysis, information on the choice of priors and Markov chain Monte Carlo settings
☒	☐	For hierarchical and complex designs, identification of the appropriate level for tests and full reporting of outcomes
☐	☒	Estimates of effect sizes (e.g. Cohen's *d*, Pearson's *r*), indicating how they were calculated

Our web collection on statistics for biologists contains articles on many of the points above.

Software and code

Policy information about availability of computer code

Data collection	No code or software was used in data collection. See below for "Data analysis".
Data analysis	Genotype data was processed using PLINK v1.9. Imputation was performed using the Michigan Imputation Server with default settings and reference data from the Haplotype Reference Consortium. All processing and analyses of methylation data was performed using R v4.0.3. Raw signal intensity data were imported into R using the minfi v1.36.0 package. For further quality control, filtering and data normalization we used the packages minfi, wateRmelon v1.26.0, CpGFilter v1.1, sva v3.38.0 and bacon v1.18.0. Linear regression was peformed using limma v3.46.0 and for meta-analyis we applied the meta v5.2-0 package. Mixed linear model-based omics association (MOA) was performed using the osca v0.46 software package. Smoking scores were estimated from methylation data using the EpiSmokEr v0.1.0 package. All analysis code used in this manuscript is available on GitHub at https://github.com/lpihlstrom/projects.

For manuscripts utilizing custom algorithms or software that are central to the research but not yet described in published literature, software must be made available to editors and reviewers. We strongly encourage code deposition in a community repository (e.g. GitHub). See the Nature Portfolio guidelines for submitting code & software for further information.

Data

Policy information about availability of data

All manuscripts must include a data availability statement. This statement should provide the following information, where applicable:

- Accession codes, unique identifiers, or web links for publicly available datasets
- A description of any restrictions on data availability
- For clinical datasets or third party data, please ensure that the statement adheres to our policy

The methylation data generated in this study have been deposited in the Gene Expression Omnibus (GEO) database under accession codes GSE203332 (NBB)

[https://www.ncbi.nlm.nih.gov/geo/query/acc.cgi?acc=GSE203332] and GSE197305 (BDR) [https://www.ncbi.nlm.nih.gov/geo/query/acc.cgi?acc=GSE197305]. Additional data on NBB donors can be obtained from the Netherlands Neurogenetics Database [https://www.brainbank.nl/nnd-project/]. Additional BDR donor data are available via the Dementias Platform UK (DPUK) data portal [https://portal.dementiasplatform.uk/]. The processed summary statistics data are available at https://github.com/lpihlstrom/projects/tree/main/Lewy_pathology_EWAS/sumstats. Other source data used to generate Figure 3 and Supplementary Figure 4 are provided in the Source Data file. The publicly available mQTL data used in this study are available from the Brain xQTLServe database [http://mostafavilab.stat.ubc.ca/xqtl/] and the SMR software homepage [https://yanglab.westlake.edu.cn/software/smr/#mQTLsummarydata]. Genotype imputation was performed using reference data available from the Haplotype Reference Consortium [http://www.haplotype-reference-consortium.org/].

Field-specific reporting

Please select the one below that is the best fit for your research. If you are not sure, read the appropriate sections before making your selection.

☒ Life sciences ☐ Behavioural & social sciences ☐ Ecological, evolutionary & environmental sciences

For a reference copy of the document with all sections, see nature.com/documents/nr-reporting-summary-flat.pdf

Life sciences study design

All studies must disclose on these points even when the disclosure is negative.

Sample size

We aimed to maximize sample size and included all samples from NBB donors meeting our inclusion criteria at the outset of the study. The BDR replication data was selected at a later stage based on identical inclusion criteria. Power calculations in epigenome-wide association studies rely on a number of uncertain assumptions, but experience indicate that the sample size we obtained could identify significant association signals, see for instance studies of similar design and comparable size in Alzheimer's disease, ref 8 and 9 respectively:
De Jager, P. L. et al. Alzheimer's disease: early alterations in brain DNA methylation at ANK1, BIN1, RHBDF2 and other loci. Nat Neurosci 17, 1156-1163 (2014)
Lunnon, K. et al. Methylomic profiling implicates cortical deregulation of ANK1 in Alzheimer's disease. Nat Neurosci 17, 1164-1170 (2014).

Data exclusions

CpG sites with a beadcount < 3 in 5% of samples or detection p-value < 0.05 in 1% of samples were filtered out using the pfilter function in the wateRmelon package. No samples had detection p-value < 5% in 5% of sites. Two samples in the NBB datset and 19 in the BDR dataset were removed due to low median signal intensities as flagged by the minfi getQC function. No outliers were detected by the wateRmelon outlyx function. Sex chromosome CpGs were used to estimate sample sex and two samples failing sex-check were removed. We estimated the proportion of NeuN positive cells in each sample using reference data from flow sorted frontal cortex cell populations as implemented in the minfi package. Two outliers samples with low NeuN proportions were removed from the NBB dataset. Probes on sex-chromosomes, probe sequences containing SNPs of any minor allele frequency in the MethylationEPIC annotation and previously reported cross-reactive probes were filtered out. It has been shown that a considerable proportion of methylation array CpGs have large measurement errors, making them unsuitable for statistical association testing in complex disorders. Taking advantage of technical replicates in the NBB dataset, we used the CpGFilter package to compute the intra-class correlation coefficient (ICC), which characterizes the relative contribution of the biological variability to the total variability for each probe, and filter out the lowest ICC quartile. Finally, the MethylSet data object was converted to beta values and the wateRmelon pwod function was used to filter out values lying more than four times the interquartile range from the mean, outliers assumed to result from rare SNP artifacts.

Replication

The study was conducted using a two-stage design. Differentially methylated loci were nominated in the NBB discovery data and reassessed in the independent BDR replication data. The main positive findings presented in the article are the associations that were significant after false discovery rate adjustment for multiple testing in the discovery stage and subsequently reached significance at two-sided p < 0.05 in the independent replication stage. Most nominated signals were not significantly replicated due to limited power, yet the overall correlation of direction and size of effects provide a strong indication of consistent results.

Randomization

Our study did not involve any intervention, so randomization was not relevant.

Blinding

Our study did not involve any intervention, so blinding of participants was not relevant. When conducting wetlab work, researchers were blinded to disease status. During data analysis we explored models using different outcomes and covariates which required that the analyst had access to phenotype data. Consequently, the analysis stage was not blinded.

Reporting for specific materials, systems and methods

We require information from authors about some types of materials, experimental systems and methods used in many studies. Here, indicate whether each material, system or method listed is relevant to your study. If you are not sure if a list item applies to your research, read the appropriate section before selecting a response.

Materials & experimental systems

n/a	Involved in the study
☒	☐ Antibodies
☒	☐ Eukaryotic cell lines
☒	☐ Palaeontology and archaeology
☒	☐ Animals and other organisms
☐	☒ Human research participants
☒	☐ Clinical data
☒	☐ Dual use research of concern

Methods

n/a	Involved in the study
☒	☐ ChIP-seq
☒	☐ Flow cytometry
☒	☐ MRI-based neuroimaging

Human research participants

Policy information about studies involving human research participants

Population characteristics	Samples in the discovery data set were obtained from the Netherlands Brain Bank (NBB, www.brainbank.nl) and Normal Aging Brain Collection, Amsterdam (NABCA). We included samples from controls without any records of neurological or psychiatric disease during life, men and women in the age range of 49-99 (see Supplementary Table 1). We included neurologically healthy donors with iLBD, clinically diagnosed and pathologically-confirmed PD patients and DLB patients. Deliberately aiming to focus our analysis on this spectrum of Lewy body disease, we excluded samples of donors with pathologically confirmed AD either alone or in addition to one of these diagnoses. For the replication stage, we used a subset of samples from the Brains for Dementia Research (BDR) cohort, men and women ranging from 41 to 104 years of age at death (see Supplementary Table 1)
Recruitment	The Netherlands Brain Bank has an extensive donor program for people with and without a brain disease. Only people who live in the Netherlands can register to be a donor. Donor recruitment and registration are organized by the NBB donor communication team. More information can be found at www.brainbank.nl/brain-tissue/donor-program/. Recruitment of donors to BDR is conducted with support from the press teams and lay representatives of Alzheimer's Research UK and the UK Alzheimers Society, using national and local press, TV and radio coverage, articles in charity newsletters, national magazines, posters, leaflets, memory clinics and public talks.
Ethics oversight	The Medical Ethics Committee of the VU University Medical Centre, Amsterdam, approved all procedures of NBB and NABCA. BDR is approved as a Research Tissue Bank by the National Research Ethics Service, UK. All participants have given informed consent. The study was approved by the Regional Committee for Health and Medical Research Ethics, Norway.

Note that full information on the approval of the study protocol must also be provided in the manuscript.
